# Comparative Evaluation of the Effectiveness of 2% Lignocaine Mixed With Dexamethasone 4 mg vs. 2% Lignocaine (1:200,000 Epinephrine) for Pain Control During the Removal of Orthodontically Indicated Mandibular Premolars: A Randomized Controlled Trial

**DOI:** 10.7759/cureus.93605

**Published:** 2025-09-30

**Authors:** Hrushikesh K Malikar, Kshitij Bang, Ramakrishna Shenoi, Vrinda Kolte, Nilima Agrawal, Rahul Dahake, Karishma D Jadhav, Teena Oommen

**Affiliations:** 1 Oral and Maxillofacial Surgery, Vidya Shikshan Prasarak Mandal (VSPM) Dental College and Research Centre, Nagpur, IND

**Keywords:** dexamethasone, local anesthesia, mandibular premolar extraction, postoperative analgesia, twin mix

## Abstract

Background

Effective pain control is paramount in dental procedures, especially during tooth extractions. While 2% lignocaine with epinephrine is the standard local anesthetic, its relatively short postoperative analgesic duration often necessitates systemic analgesics. Incorporating dexamethasone, a potent anti-inflammatory corticosteroid, into local anesthetic solutions has shown promise in enhancing anesthetic efficacy and postoperative comfort.

Materials & methods

This double-blind, split-mouth randomized controlled trial enrolled 30 healthy volunteers undergoing bilateral mandibular premolar extractions for orthodontic purposes. Each extraction site was randomly assigned to one of two protocols in a single session: group A (twin mix) received a mixture of 1.8 mL 2% lignocaine with 1:200,000 epinephrine plus 1 mL of 4 mg dexamethasone, while group B (standard control) received only 1.8 mL of the same lignocaine-epinephrine formulation. In total, 60 extraction sites were included, and each patient served as their own control.

Results

The results demonstrated that the twin mix offered clinical advantages: patients in the twin mix group reported less pain during anesthetic injection (mean: 3.57 vs. 6.43; p < 0.001), experienced faster anesthesia onset (14.0 vs. 24.3 seconds; p < 0.001), and had enhanced duration of anesthesia (111.7 vs. 73.1 minutes; p < 0.001). There was no significant difference in pain during tooth extraction (p = 0.302). Notably, 26 (86.7%) of twin mix patients required no postoperative analgesics compared to 23 (76.7%) in the control group. These findings align with prior studies showing that dexamethasone added to lignocaine accelerates the onset, prolongs anesthesia, and reduces pain and analgesic consumption.

Conclusion

The twin mix enhanced anesthesia by reducing injection pain, accelerating onset, prolonging the duration of action, and decreasing postoperative analgesic use, supporting its value as a superior, patient-centered option for minor oral surgery.

## Introduction

Effective pain control is essential in dental procedures. The removal of orthodontically indicated mandibular premolars is a common practice in orthodontic treatment planning, often undertaken to relieve crowding, correct dental protrusion, or align the dental arches for optimal occlusion [1]. Although these procedures are generally straightforward and minimally invasive, they still require efficient and reliable local anesthesia to ensure patient comfort, cooperation, and procedural success, especially since the majority of patients undergoing such extractions are young and often anxious [2].

Local anesthesia works by reversibly blocking voltage-gated sodium channels at the injection site, stopping nerve signals and enabling painless dental procedures. Commonly used amide anesthetics, such as 2% lignocaine, are formulated with epinephrine (1:100,000 or 1:200,000) to constrict blood vessels, which slows drug absorption, prolongs duration of action, and reduces bleeding. The current research prioritizes formulations that offer faster onset, deeper anesthesia, and longer duration with minimal side effects [3,4].

Studies have shown that adding dexamethasone to lignocaine can extend anesthesia duration, reduce postoperative pain and swelling, and enhance patient recovery [5,6]. Dexamethasone, a powerful synthetic corticosteroid, is widely recognized for its anti-inflammatory and immunosuppressive properties. In dental surgeries, particularly the extraction of impacted mandibular third molars, it plays a crucial role in managing postoperative complications such as pain, swelling, and trismus. When administered either locally or systemically, dexamethasone effectively reduces these postoperative sequelae, enhancing patient comfort and recovery. An innovative approach in dental anesthesia is the "twin mix," which combines 2% lignocaine with 1:200,000 epinephrine and 4 mg dexamethasone in a single injection. This mixture not only provides effective local anesthesia but also provides the anti-inflammatory benefits of dexamethasone. Clinical studies have demonstrated that the twin mix offers a faster onset, prolongs the duration of anesthesia, and improves postoperative outcomes compared to traditional lignocaine-epinephrine solutions. Patients receiving the twin mix report reduced postoperative discomfort, swelling, and improved mouth opening, indicating its efficacy in enhancing the quality of dental care [6,7].

Given the rising interest in enhancing anesthetic techniques for routine dental extractions, the “twin mix” offers a promising alternative to conventional local anesthesia. Although dexamethasone has demonstrated significant benefits in managing postoperative discomfort in oral surgeries, its combined use with lignocaine for orthodontically indicated premolar extractions remains underexplored. There is currently no consensus on its superiority over standard lignocaine with epinephrine formulations. This gap in evidence emphasizes the need for well-designed randomized controlled trials to determine whether twin mix offers clinically meaningful advantages in terms of pain control, onset, duration, and postoperative recovery. Therefore, the aim of this study is to compare the efficacy of twin mix versus 2% lignocaine hydrochloride with 1:200,000 epinephrine for pain control during the removal of orthodontically indicated mandibular premolars.

## Materials and methods

Study design

**Figure 1:**
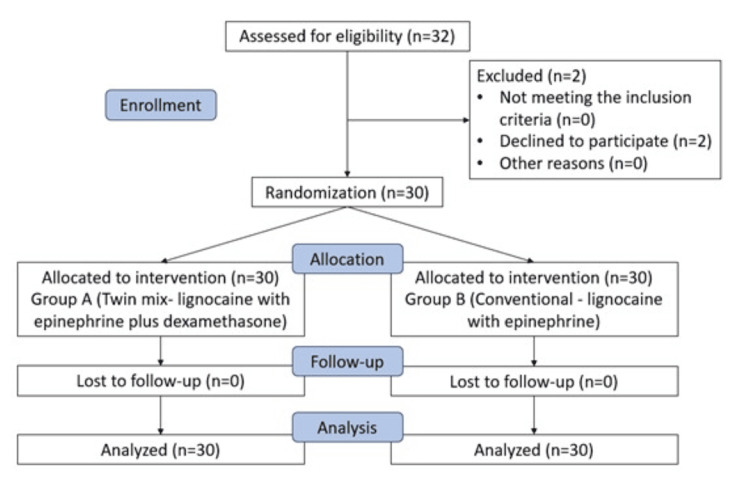
CONSORT flowchart. CONSORT: Consolidated Standards of Reporting Trials.

This randomized, split-mouth, double-blinded controlled clinical trial was conducted in the Department of Oral and Maxillofacial Surgery following approval from the Institutional Ethics Committee, Vidya Shikshan Prasarak Mandal (VSPM) Dental College and Research Centre, in accordance with the CONSORT guidelines. The study was registered under the Clinical Trial Registry - India (CTRI/2025/07/091212). Healthy individuals who required bilateral extraction of mandibular premolars as part of their orthodontic treatment plan were enrolled in the study (Figure 1).

Inclusion and exclusion criteria

The inclusion criteria comprised patients aged above 15 years of either gender, who were systemically healthy, consenting to participate in the study, and maintained good oral hygiene. Patients were excluded if they were pregnant or lactating, had periapical infections in the mandibular premolars, were allergic to local anesthetics or anti-inflammatory drugs, had high-risk systemic conditions, or declined to give consent or attend follow-up appointments.

Randomization and allocation process

The sites and allocated treatment were randomized as per the simple block randomization list generated by sealed envelope software. Upon patient enrolment and after obtaining written informed consent, an envelope was opened to reveal the site assignment for each group.

**Figure 2:**
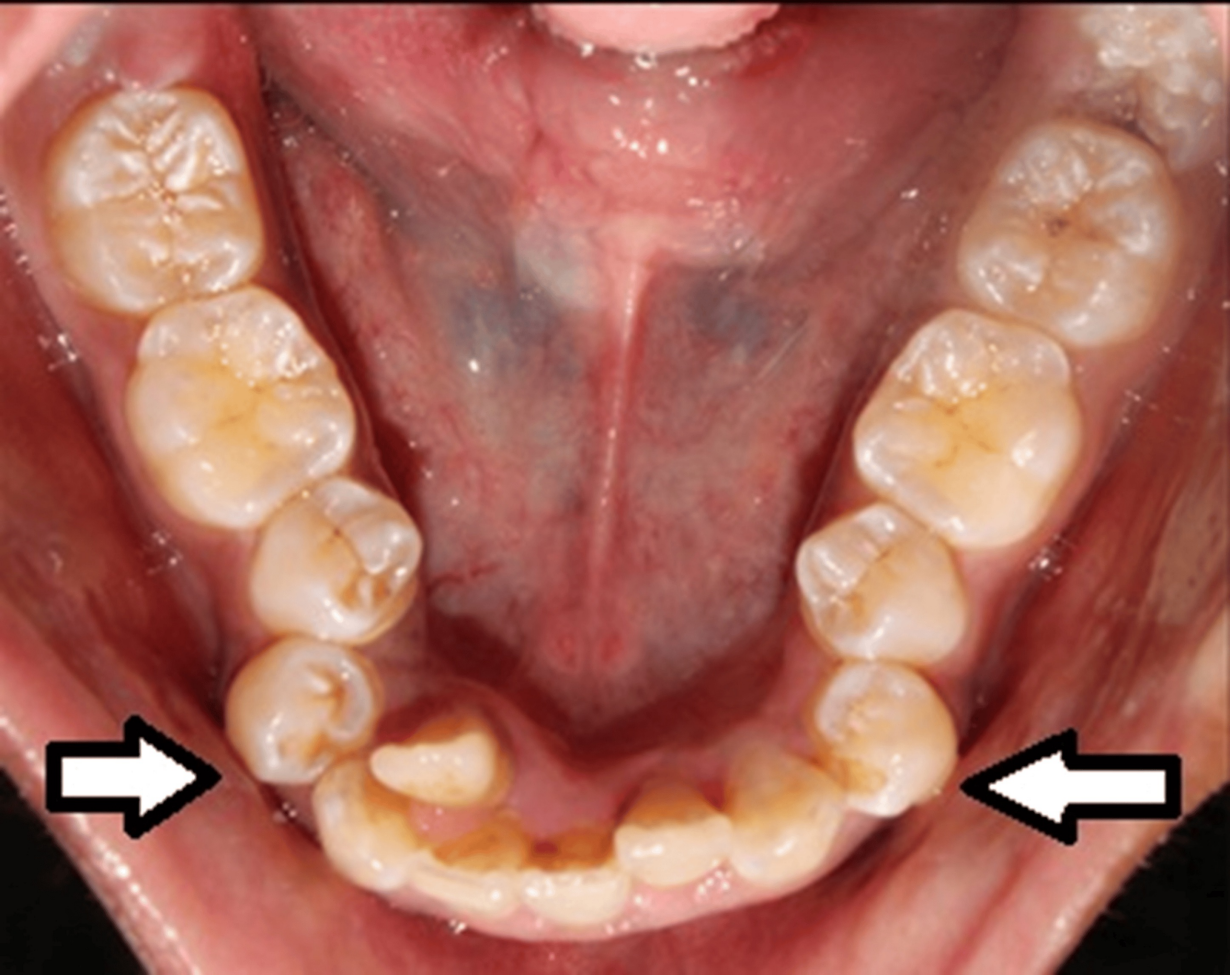
Patient indicated for bilateral orthodontic extraction of mandibular premolars. Image credit: Dr. Hrushikesh Malikar.

A total of 30 healthy individuals who volunteered for this split-mouth study were enrolled. Since each patient required bilateral mandibular premolar extractions, a total of 60 extraction sites were obtained (Figure 2).

Each patient acted as their own control: one extraction site was randomly assigned to group A (twin mix - 1.8 ml of 2% lignocaine with 1:200,000 epinephrine plus 1 ml of 4 mg dexamethasone), and the contralateral site to group B (conventional - 2% lignocaine with 1:200,000 epinephrine) was treated one week later to maintain uniformity in the timing of interventions.

Preoperative assessment

A comprehensive case history was recorded preoperatively using a standardized case history form, documenting past medical history and any known allergies, especially to local anesthetic agents. Preoperative orthopantomogram radiographs were taken for all patients to assess the teeth indicated for extraction. Hematological investigations, including random blood sugar, bleeding time, and clotting time, were performed. Cases where abnormal values were observed were excluded from the study.

Surgical procedure

All procedures were carried out under strict aseptic conditions. The inferior alveolar nerve block (IANB) was administered following a standardized technique, which includes positioning the patient in a semi-reclined posture with the mouth wide open. A 25-gauge long needle is inserted on the medial side of the mandibular ramus, lateral to the pterygomandibular raphe, at the height of the coronoid notch. The needle is advanced until bone is contacted near the mandibular foramen, then withdrawn slightly, aspirated to avoid intravascular injection, and 1.5-1.8 mL of anesthetic solution is slowly deposited [2].

The extraction was performed on one side with either group A or group B, depending on the randomization, and the contralateral side was treated one week later to maintain uniformity in the timing of interventions.

All extractions were performed by the same operator to ensure procedural consistency. Hemostasis was achieved postoperatively, and standard postoperative care instructions were given.

Parameters assessed

Parameters assessed in this study were pain during the deposition of anesthesia, onset and duration of anesthesia, pain during tooth extraction, and postoperative analgesic requirement. Pain during both the administration of anesthesia and the extraction procedure was recorded using a 10-point visual analog scale (VAS), where patients were rated for their discomfort on a scale from 0 (no pain) to 10 (worst pain) [2].

The onset of anesthesia was measured using a stopwatch, starting from the time of injection until the patient reported numbness in the lower lip, indicating effective nerve blockade. The duration of anesthesia was noted from the onset of action to the point when the sensation returned. The need for postoperative analgesics was documented, with a score of 1 indicating the requirement for pain relief and 0 if none was needed.

Statistical analysis

Data were analyzed using IBM SPSS Statistics version 20 (IBM Corp., Armonk, NY). Normality was checked with the Shapiro-Wilk test. Since the data were normally distributed, results were expressed as mean and standard deviation. An independent t-test was used to compare quantitative variables between group A and group B. A paired t-test assessed within-group differences in pain during anesthesia and extraction. A p-value < 0.05 was considered statistically significant.

## Results

In the current study, out of 30 patients, 18 were female and the remaining 12 were male, with a mean age of 19 years.

Table 1 presents the findings from the independent t-test comparing four key clinical parameters between group A (twin mix) and group B (conventional lignocaine with epinephrine).

**Table 1:** Comparison of pain during the deposition of anesthesia, onset of anesthesia, pain during tooth extraction, and duration of anesthesia between group A and group B.

Parameters	Mean ± Std. deviation		t-value	P-value	Significance
	Group A	Group B			
Pain during the deposition of anesthesia	3.57±0.817	6.43±0.935	-12.642	0.000	Significant
Onset of anesthesia (in seconds)	14.03±2.606	24.30±5.902	-8.715	0.000	Significant
Pain during the extraction of a tooth	4.73±7.254	6.13±1.306	-1.04	0.302	Not significant
Duration of anesthesia (in minutes)	111.70±18.334	73.07±9.24	10.307	0.000	Significant

Differences were observed in three out of the four parameters assessed. Pain during the deposition of anesthesia was lower in group A (mean = 3.57 ± 0.817) compared to group B (mean = 6.43 ± 0.935), with a t-value of -12.642 and a p-value of <0.001, indicating a statistically significant reduction in pain in the test group. The onset of anesthesia occurred faster in group A (14.03 ± 2.606 seconds) than in group B (24.3 ± 5.902 seconds), as reflected by a t-value of -8.715 and a p-value < 0.001. Similarly, the duration of anesthesia was considerably longer in group A (111.7 ± 18.334 minutes) than in group B (73.07 ± 9.24 minutes), with a t-value of 10.307 and p-value < 0.001, showing statistical significance. However, for pain experienced during tooth extraction, no statistically significant difference was observed between group A (4.73 ± 7.254) and group B (6.13 ± 1.306). The t-value of -1.04 and p-value of 0.302 indicate that pain levels during tooth removal were comparable between the groups.

Table 2 summarizes the postoperative analgesic requirement in both groups. A total of 26 patients (86.7%) in group A did not require additional analgesia after the procedure, compared to 23 (76.7%) in group B.

**Table 2:** Comparison of the need for postoperative analgesia between group A and group B.

Variables		Group		Total
		A, n (%)	B, n (%)	
Need for postoperative analgesia	No	26 (86.7)	23 (76.7)	49 (81.7)
	Yes	4 (13.3)	7 (23.3)	11 (18.3)
Total		30 (100)	30 (100)	60 (100)

Conversely, the percentage of patients who required postoperative analgesics was higher in group B (23.3%) than in group A (13.3%), suggesting that patients in group A experienced better postoperative pain control.

## Discussion

Local anesthesia functions by reversibly blocking voltage-gated sodium channels in nerve fibers, preventing pain signals from reaching the central nervous system. In dentistry, amide anesthetics, such as lignocaine, articaine, and bupivacaine, are preferred due to their reliable effect and low allergy risk. When combined with vasoconstrictors like epinephrine, their efficacy is enhanced: absorption is slowed, duration is extended, and bleeding at the injection site is minimized [8]. The IANB is the standard technique for mandibular procedures, anesthetizing the lower teeth, lip, and adjacent tissues by targeting the mandibular nerve before it enters the foramen [9]. Orthodontic premolar extractions, performed to correct alignment and occlusion, require effective anesthesia. The conventional practice uses 2% lignocaine with epinephrine; however, postoperative pain remains a concern. This study compared the standard approach against the “twin mix,” combining lignocaine, epinephrine, and dexamethasone, a corticosteroid known to suppress inflammatory mediators like prostaglandins and leukotrienes, to assess improvements in anesthetic quality. Dexamethasone has been shown to extend duration, reduce pain and swelling, and hasten onset when used in oral surgery [10].

The present study demonstrates that the “twin‑mix” formulation, combining lignocaine with dexamethasone, offers significant clinical advantages over standard lignocaine-adrenaline anesthesia. Adding dexamethasone to lignocaine with epinephrine slightly raises the solution’s pH, making it less acidic. This buffering reduces injection pain, may speed up anesthetic onset, and provides anti-inflammatory and analgesic effects, helping to minimize postoperative pain and inflammation. Specifically, patients receiving twin mix reported markedly lower pain scores during injection (3.57 ± 0.82 vs. 6.43 ± 0.94, P = 0.000), paralleling Bhargava et al. (2013), who showed that perineural dexamethasone reduced latency and improved comfort during mandibular third‑molar surgery [10]. Furthermore, twin‑mix achieved a faster onset of action (14.0 seconds vs. 24.3 seconds, P = 0.000) and extended anesthesia duration (111.7 minutes vs. 73.1 minutes, P = 0.000), consistent with findings from Sahu et al. (2020) and Poorna et al. (2024), where the addition of dexamethasone significantly shortened onset time and prolonged block duration in mandibular nerve blocks [11,12]. Notably, the twin‑mix group also required less postoperative analgesia; 86.7% did not need further pain relief compared to 76.7% in the control group, a finding that aligns with Beena et al. (2020), who reported decreased analgesic consumption following steroid‑augmented local anesthesia in third‑molar extraction [13].

Interestingly, pain during tooth extraction did not significantly differ between groups (P = 0.302), echoing Poorna et al. (2024), which suggests that once mechanical forces are applied, both solutions are equally effective under clinical manipulation. This indicates that the enhanced benefits of dexamethasone are most apparent during the injection and early postoperative periods, rather than during the extraction itself [12]. Finally, neither group experienced postoperative trismus, likely a consequence of the less invasive premolar extractions used in this study compared to third‑molar surgeries, where trismus and swelling are common, similar to observations in Bhargava et al. (2013) and twin‑mix literature [10].

The study had several limitations. First, it focused solely on mandibular premolar extractions, which limits the generalizability of the results to other dental or oral surgical procedures. Second, since the twin mix must be prepared chair-side rather than supplied as a standardized commercial product, variations in its preparation could affect consistency and outcomes. Third, the study did not assess long-term effects, such as tissue healing, delayed wound closure, or corticosteroid-related complications like hyperglycemia or infection risk. Finally, the underlying biochemical mechanisms behind dexamethasone’s enhancement of anesthetic effects, as well as its interactions with other adjuvants, were not explored, highlighting an important area for further study.

## Conclusions

Overall, these findings suggest that the use of twin mix significantly enhances patient comfort by reducing injection pain, accelerating onset, prolonging anesthesia, and decreasing postoperative analgesic demand, although it does not impact extraction-phase discomfort. These benefits highlight the twin‑mix formulation as a promising option for routine minor oral surgical procedures. Future studies should explore its applicability in more complex extractions and broader clinical settings to further validate its benefits.
